# Risk stratification of oesophageal squamous cell carcinoma using change in total lesion glycolysis and number of PET-positive lymph nodes

**DOI:** 10.1038/s41416-023-02151-y

**Published:** 2023-02-25

**Authors:** Yohei Nose, Tomoki Makino, Mitsuaki Tatsumi, Koji Tanaka, Kotaro Yamashita, Toshiki Noma, Takuro Saito, Kazuyoshi Yamamoto, Tsuyoshi Takahashi, Yukinori Kurokawa, Kiyokazu Nakajima, Hidetoshi Eguchi, Yuichiro Doki

**Affiliations:** 1 Department of Gastroenterological Surgery, Graduate School of Medicine, Osaka University, Suita, Japan; 2 Department of Radiology, Graduate School of Medicine, Osaka University, Suita, Japan

**Keywords:** Oesophageal cancer, Chemotherapy

## Abstract

**Background:**

The efficacy of neoadjuvant chemotherapy (NACT) correlates with patient survival in oesophageal squamous cell carcinoma (OSCC), but optimal evaluation of the treatment response based on PET-CT parameters has not been established.

**Methods:**

We analysed 226 OSCC patients who underwent PET-CT before and after NACT followed by surgery. We assessed SUVmax, metabolic tumour volume (MTV), and total lesion glycolysis (TLG) for the primary tumour and the number of PET-positive lymph nodes before and after NACT to predict patient survival.

**Results:**

In a stepwise analysis, we defined 60%, 80%, and 80% as the optimal cut-off values for SUVmax, MTV, and TLG reduction, respectively, to distinguish responders and non-responders to NACT. In the ROC analysis, the TLG reduction rate was the best predictor of recurrence among PET-CT parameters. The TLG responders achieved significantly more favourable prognoses than non-responders (2-year progression-free survival [PFS] rate: 64.1% vs. 38.5%; *P* = 0.0001). TLG reduction rate (HR 2.58; 95% CI 1.16–5.73) and the number of PET-positive lymph nodes after NACT (HR 1.79; 95% CI 1.04–3.08) were significant independent prognostic factors.

**Conclusions:**

TLG reduction is the best predictor of prognosis. Preoperative PET-CT evaluation of both the primary tumour and lymph nodes could accurately stratify risk in OSCC patients.

## Introduction

Oesophageal cancer (OC) is the sixth most common cause of cancer-related death worldwide and is a major global health challenge [1]. Neoadjuvant chemotherapy (NACT) is generally used for locally advanced oesophageal squamous cell carcinoma (OSCC) [2]. The response of the primary tumour to NACT is of great prognostic importance [3–5], and evaluation by positron emission tomography-computed tomography (PET-CT) has been reported to be useful in developing treatment strategies for OSCC [6, 7]. A relationship has been reported between the standardised uptake value (SUV), a commonly used parameter for semi-quantitative analysis of PET-CT images, and the prognosis and treatment response in OC [8, 9]. However, the SUV is influenced by several factors, including body composition and habitus, length of uptake period, plasma glucose, and partial volume effects. Furthermore, maximum SUV (SUVmax) does not represent the whole tumour [10–12].

In contrast, tumour volume indices that take into account metabolic activity, such as metabolic tumour volume (MTV) and total lesion glycolysis (TLG), have been attracting attention as new indices for PET-CT [13]. MTV is measured by contouring margins defined by thresholds, whereas TLG is calculated by multiplying MTV by the mean SUV (SUVmean) [14]. The use of MTV and TLG has been proposed to assess disease burden and tumour invasiveness by quantifying the metabolic volume burden and activity of tumours [15]. Several reports have been published on the relationship between these PET-CT parameters before and after chemoradiotherapy (CRT) and the prognosis of OC [16–21]. However, evidence of PET-CT parameters, particularly volumetric ones, before and after NACT being associated with OC patient survival is limited [22, 23].

Therefore, we aimed to investigate the utility of measuring SUVmax, MTV, and TLG values of the primary tumour in addition to the number of positive lymph nodes (LNs) using PET-CT before and after NACT as indicators of treatment efficacy and prognosis in patients with locally advanced OSCC.

## Methods

### Patient eligibility

This retrospective study included 285 consecutive patients with thoracic OC without distant metastasis who underwent surgery after NACT at Osaka University Hospital from January 2010 to December 2016. Among these patients, 233 had histologically confirmed squamous cell carcinoma and underwent PET-CT before and after NACT. With the availability of data on PET-CT parameters (SUVmax, MTV, and TLG), 226 cases were finally analysed after excluding 7 with SUVmax values <2.5 in the primary tumour before NACT. Patients with cervical or coeliac LN metastasis were eligible for inclusion. All patients were staged according to UICC criteria before and after surgery. Clinical staging before NACT was based on esophagography, endoscopy, and computed tomography (CT) of the neck, chest, and upper abdomen using continuous 5-mm-thick slices. All patients had adequate cardiac, hepatic, renal, and bone marrow reserves and could tolerate both the NACT and surgery. This study was approved by the Human Ethics Review Committee of the Osaka University Graduate School of Medicine (Osaka, Japan, approval number; 08226), and signed consent for participation and publication was obtained from each patient.

### Surgical treatment

Our standard surgical procedures comprised subtotal esophagectomy with mediastinal lymphadenectomy via right thoracotomy, upper abdominal lymphadenectomy, gastric tube reconstruction, and anastomosis in the cervical incision [6, 7, 24]. A three-field lymphadenectomy was performed for patients with supraclavicular or recurrent laryngeal nerve lymph node metastases on preoperative staging or intraoperative diagnosis, and patients with a primary tumour located in the upper third of the thoracic oesophagus [25]. The remaining patients underwent a two-field lymphadenectomy.

### 18F-FDG PET-CT acquisition and analysis

All patients underwent 18F-fluorodeoxyglucose (18F-FDG) PET-CT before and within 2–3 weeks after completion of NACT as described previously [6, 7, 22, 26]. 18F-FDG PET-CT was performed with an integrated scanner (Gemini GXL; Philips, Amsterdam, the Netherlands), and whole-body images, generally from the top of the skull to mid-thigh, were acquired approximately 60 min after intravenous injection of [^18^F]-FDG at a dose of 3.7 MBq (0–10 mCu) per kilogram of body weight. Regions of interest (ROIs) were placed over the areas of the primary tumours with maximum FDG uptake on the baseline scans for semi-quantitative analysis. The SUVmax was calculated using previously reported methods [26], and MTV was defined as the tumour volume with SUV >2.5 [22]. TLG was defined as the SUVmean multiplied by MTV. Semi-quantitative and volumetric analyses of the primary tumours were performed to measure the PET-CT parameters reported in the present study using the volume viewer software SYNAPSE VINCENT^®^ (Fujifilm Medical, Tokyo, Japan), which can easily delineate a volume of interest (VOI) with an iso-contour threshold method based on the SUV. The software automatically calculates SUVmax, MTV, and TLG within the entire primary tumour when a spherical VOI is drawn to encompass the primary lesion (Supplemental Fig. 1). SUVmax, MTV, and TLG were measured before and after NACT in all patients, and the reduction of each parameter was calculated and included in the analysis. LNs with SUVmax ≥2.5 by PET-CT were considered positive. All assessments were performed by one radiologist and more than two surgeons that specialise in OC.

### Neoadjuvant chemotherapy

Our hospital adopted the NACT regimen comprising triplet chemotherapy with either 5-fluorouracil (5-FU), cisplatin, and doxorubicin (FAP), or 5-FU, cisplatin, and docetaxel (DCF) as described previously [27–30]. For the FAP regimen, 700 mg/m^2^ 5-FU was given by continuous intravenous infusion on days 1–7, along with 70 mg/m^2^ cisplatin by intravenous infusion, and 35 mg/m^2^ doxorubicin by rapid intravenous infusion on day 1 [31–33]. For the DCF regimen, cisplatin was administered at 70 mg/m^2^, docetaxel at 70 mg/m^2^ by rapid intravenous infusion on day 1, and 5-FU at 700 mg/m^2^ by continuous intravenous infusion on days 1–5 [31]. With either regimen, two courses of NACT were usually used at a 3–4-week interval. Other regimens used in the present study included 5-FU plus cisplatin (5-FU, 800 mg/m^2^/day, days 1–5; cisplatin, 80 mg/m^2^/day, day 1, repeated every 3 weeks) for three patients and nedaplatin plus paclitaxel for one patient [34].

### Evaluation of clinical response

All patients were re-staged by CT, endoscopy, and PET-CT to evaluate the clinical response 2 to 3 weeks after the completion of NACT [22, 35]. The response was categorised based on the World Health Organisation response criteria for measurable disease and the Japanese Society for Oesophageal Disease criteria. Histopathological findings were classified according to UICC TNM classifications, and the degree of histopathological tumour regression in the surgical specimens was classified into five categories. The extent of viable residual carcinoma at the primary tumour was assessed semi-quantitatively based on the estimated percentage of viable residual carcinoma about the macroscopically identifiable tumour bed that was evaluated histopathologically. The percentage of viable residual tumour cells within the entire cancerous tissue was assessed as follows: grade 0, no significant response to NACT; grade 1a, more than two-thirds of residual tumour cells; grade 1b, one-third to two-thirds of residual tumour cells; grade 2, less than one-third of residual tumour cells; grade 3, no viable residual tumour cells [36, 37]. The severity of postoperative complications was evaluated according to the Clavien–Dindo classification system [38]. Progression-free survival (PFS) was defined as the time from surgery to either the first recurrence or death from any cause. Overall survival (OS) was defined as the time from surgery to death from any cause.

### Statistical analysis

We used the Student’s *t* test to compare the averages of continuous variables between two groups, and the Mann–Whitney *U*-test, chi-squared test, and Fisher’s exact test to compare the proportions of categorical variables. Receiver operating characteristic (ROC) curve analysis was applied to identify the best discriminating cut-off values for SUVmax, MTV, and TLG. A multivariate Cox proportional hazard regression model with stepwise comparisons was used to identify independent prognostic markers. Prognostic variables were assessed by the log-rank test, and PFS and OS were analysed by the Kaplan–Meier method. Univariate and multivariate analyses of clinico-pathological variables were used to compare the reliability of the PET-CT parameters. *P* < 0.05 indicated significant differences. All statistical analyses were carried out using JMP^®^14 (SAS Institute Inc, Cary, NC, USA).

## Results

### Patient characteristics

The patient characteristics are summarised in Supplemental Table 1. Twenty-three patients had cM1 disease, which was confined to metastasis of the supraclavicular LNs. All 226 patients underwent surgical resection after NACT. The most common NACT regimen was DCF (69.5%), followed by FAP (28.8%). Curative (R0) surgery was performed for the majority of patients (97.3%) while six patients (2.7%) received R1 resection. Three-field lymphadenectomy was performed in 129 cases (57.1%).

### PET-CT evaluation of primary tumour and lymph nodes

The median SUVmax, MTV, and TLG values for the primary tumour before NACT were 12.2 (2.8–40.9), 19.2 (0.4–232.7), and 96.3 (1.2–1619.3), respectively, and after NACT were 3.4 (1.5–17.9), 1.6 (0–52.9), and 4.5 (0–345.8), respectively. All three PET-CT parameters were significantly lower after NACT (each *P* < 0.0001). The number of PET-positive LNs before NACT (pre-NACT PET-N) was 0, 1, and >2 in 93 (41.2%), 49 (21.7%), and 84 (37.2%) cases, respectively, and the number of PET-positive LNs after NACT (post-NACT PET-N) was 0, 1, and >2 in 161 (71.2%), 33 (14.6%), and 32 (14.2%) cases, respectively.

### Optimal cut-off values based on survival analysis

We conducted a stepwise analysis to determine the optimal cut-off for each parameter that clearly discriminates between responders and non-responders to NACT based on PFS. We achieved this by evaluating cut-off values for each PET-CT parameter at each 10% reduction from 0% to 90% (Table 1). The cut-off values of 60%, 80%, and 80% for the SUVmax, MTV, and TLG reduction rate, respectively, showed the largest difference in PFS between responders and non-responders and the lowest *P* values (SUVmax: hazard ratio [HR] = 1.75, *P* = 0.0026; MTV: HR = 1.70, *P* = 0.0054; TLG: HR = 2.04, *P* = 0.0003). Therefore, we defined 60%, 80%, and 80% as the optimal cut-off values of the reduction in SUVmax, MTV, and TLG, respectively, to separate responders and non-responders to NACT for locally advanced OSCC.

**Table 1:** Stepwise regression analysis for the identification of optimal each parameter reduction rate cut-off value (*n* = 226).

*SUVmax reduction rate, %*	10	20	30	40	50	**60**	70	80	90
Number									
Responders	218	203	190	166	145	**119**	86	45	1
Non-responders	8	23	36	60	81	**107**	140	181	225
5-year PFS rate, %									
Responders	50.2	51.0	50.6	54.9	56.1	**60.0**	59.9	54.6	100
Non-responders	37.5	39.1	46.3	35.5	38.5	**38.4**	43.5	48.6	49.6
* P* value	0.45	0.066	0.29	0.0028	0.0081	**0.0026**	0.021	0.77	0.24
Hazard ratio	1.44	1.72	1.31	1.84	1.66	**1.75**	1.57	1.07	-
*MTV reduction rate, %*	10	20	30	40	50	60	70	**80**	90
Number									
Responders	217	216	209	205	194	183	165	**144**	124
Non-responders	9	10	17	21	32	43	61	**82**	102
5-year PFS rate, %									
Responders	51.1	50.9	51.6	51.8	52.2	53.8	53.0	**56.6**	56.0
Non-responders	14.8	25.0	26.9	30.2	34.7	32.1	41.2	**37.8**	42.3
* P* value	0.066	0.15	0.035	0.037	0.043	0.0066	0.078	**0.0054**	0.031
Hazard ratio	2.23	1.84	2.03	1.91	1.70	1.86	1.44	**1.70**	1.49
*TLG reduction rate, %*	10	20	30	40	50	60	70	**80**	90
Number									
Responders	218	218	214	212	203	194	179	**160**	135
Non-responders	8	8	12	14	23	32	47	**66**	91
5-year PFS rate, %									
Responders	50.9	50.9	51.4	51.9	51.7	53.6	54.3	**57.1**	58.1
Non-responders	18.8	18.8	20.8	17.1	32.8	26.6	32.1	**32**	37.4
* P* value	0.079	0.079	0.043	0.012	0.11	0.0023	0.0049	**0.0003**	0.0018
Hazard ratio	2.30	2.30	2.19	2.45	1.61	2.17	1.85	**2.04**	1.79

*PFS* progression-free survival, *MTV* metabolic tumour volume, *TLG* total lesion glycolysis.

Bold values indicate the optimal cut-off values of the reduction in SUVmax, MTV, and TLG showing the largest survival difference between responders and non-responders and the lowest *P* values.

**Fig. 1:**
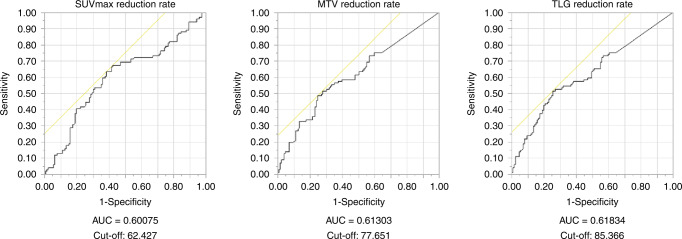
Receiver operating characteristic (ROC) analysis of postoperative recurrence. The area under the curve (AUC) and cut-off for each PET-CT indicator are shown.

We performed a ROC analysis of disease recurrence to determine the optimal cut-off value for the reduction of three PET-CT parameters as shown in Fig. 1. The area under the curve (AUC) identified the TLG reduction to be the largest among the parameters. For all parameters, the cut-off values obtained by the stepwise method were the approximate values of those determined by the ROC analysis.

### Clinico-pathological parameters associated with PET-CT evaluation

Using the cut-off value for the reduction in TLG determined by the stepwise method, we divided all patients into two groups: TLG responders (TLG reduction rate ≥80%) and TLG non-responders (TLG reduction rate <80%). We then compared various clinico-pathological factors as shown in Table 2. We did not find any significant differences between the two groups in terms of age, sex, tumour location, tumour size, differentiation, pre-therapeutic PET-CT value, clinical TNM status, residual tumour, pre- and post-NACT PET-N, or postoperative complication. Compared to the TLG non-responders, TLG responders had a higher proportion of patients receiving the DCF regimen (77.5% vs. 50.0%; *P* < 0.0001), better histological response (Grade 1b-3; 72.5% vs. 45.5%; *P* = 0.0001), and less advanced pT stage (pT0-2; 65.0% vs. 30.3%; *P* < 0.0001; Table 2).

**Table 2:** Correlation between TLG reduction rate and clinico-pathological parameters.

	TLG reduction rate ≥80%	TLG reduction rate <80%	*P* value
	(*n* = 160)	(*n* = 66)	
Median age (range)	67 (38–81)	66 (35–82)	0.63
Sex			
Male	137 (85.6%)	60 (90.9%)	0.28
Female	23 (14.4%)	6 (9.1%)	
Tumour location			
Upper	27 (16.9%)	8 (12.1%)	0.37
Middle, lower	133 (83.1%)	58 (87.9%)	
Tumour size^a^ (range)	500 (45–3976)	528 (86–1832)	0.3
Differentiation			
Well differentiated	27	16	0.52
Moderately differentiated	99	40	
Poorly differentiated	13	7	
Unknown	21	3	
Pre-therapeutic PET-CT value			
SUVmax	12.5 (2.82–40.91)	11.9 (2.89–28.41)	0.49
MTV	19.1 (0.5–232.7)	19.3 (0.4–84.9)	0.89
TLG	97.8 (1.4–1619.3)	85.6 (1.2–511.4)	0.79
Preoperative chemotherapy			
DCF	124 (77.5%)	33 (50.0%)	**<0.0001**
Others	36 (22.5%)	33 (50.0%)	
cT			
T1–2	50 (31.3%)	15 (22.7%)	0.19
T3–4	109 (68.7%)	51 (77.3%)	
cN			
N0	40 (25.0%)	22 (33.3%)	0.2
N1	120 (75.0%)	44 (66.7%)	
cM lym	17 (10.6%)	6 (9.1%)	0.73
Histological response			
Grade 0–1a	44 (27.5%)	36 (54.5%)	**0.0001**
Grade 1b–3	116 (72.5%)	30 (45.5%)	
Residual tumour			
R0	157 (98.1%)	63 (95.5%)	0.26
R1	3 (1.9%)	3 (4.5%)	
Number of PET-positive LNs before NACT			0.44
0–1	98 (61.3%)	44 (66.7%)	
≥2	62 (38.8%)	22 (33.3%)	
Number of PET-positive LNs after NACT			0.49
0–1	139 (86.9%)	55 (83.3%)	
≥2	21 (13.1%)	11 (16.7%)	
Postoperative complication^b^			
0–II	119 (74.4%)	44 (66.7%)	0.24
III–V	41 (25.6%)	22 (33.3%)	
pT			
T0–2	104 (65.0%)	20 (30.3%)	**<0.0001**
T3–4	56 (35.0%)	46 (69.7%)	
pN			
N0	59 (36.9%)	16 (24.2%)	0.067
N1	101 (63.1%)	50 (75.8%)	
pM			
M0	151 (94.4%)	60 (90.9%)	0.34
M1	9 (5.6%)	6 (9.1%)	

*MTV* metabolic tumour volume, *TLG* total lesion glycolysis, *DCF* docetaxel, cisplatin, and 5-fluorouracil, *Others* 5-fluorouracil, cisplatin, and doxorubicin/5-fluorouracil and cisplatin/nedaplatin and paclitaxel, *LN* lymph node, *NACT* neoadjuvant chemotherapy.

^a^Tumour size: major axis × minor axis (mm).

^b^Postoperative complication: classified by Clavien–Dindo classification.

Bold values indicate *P* values with stratistical significance.

### Prognostic significance of PET-CT evaluation

**Fig. 2:**
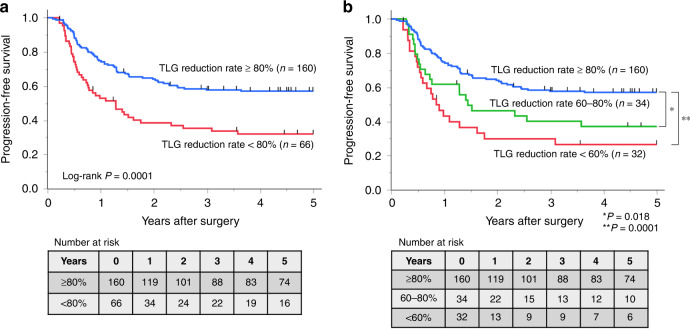
Progression-free survival (PFS) classified by the total lesion glycolysis (TLG) reduction rate in the primary tumour during neoadjuvant chemotherapy. **a** The TLG responders (TLG reduction rate ≥80%) achieved significantly more favourable prognoses than non-responders (TLG reduction rate <80%). Two-year PFS rate: 64.1 vs. 38.5%, *P* = 0.0001. **b** When classified into three groups according to TLG reduction rate (80–100%, 60–80%, <60%), 2-year PFS rates were 64.1%, 46.3%, and 29.9%, respectively.

**Fig. 3:**
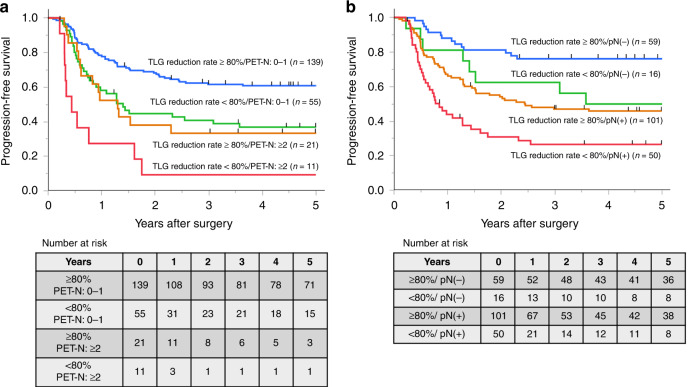
Kaplan–Meier analysis of progression-free survival (PFS). **a** For all patients classified into four groups based on the total lesion glycolysis (TLG) reduction rate and number of post-NACT PET-positive lymph nodes (PET-N). **b** For all patients classified into four groups based on TLG reduction rate and pN.

The median follow-up time was 4.51 (range, 0.22–10.88) years. The TLG responders achieved significantly more favourable prognoses than non-responders (2-year PFS rate: 64.1% vs. 38.5%, *P* = 0.0001, Fig. 2a; 2-year OS rate: 84.0% vs. 56.8%; *P* < 0.0001). When classified into three groups according to TLG reduction rate (80–100%, 60–80%, <60%), the 2-year PFS rates were 64.1%, 46.3%, and 29.9%, respectively (Fig. 2b). Univariate analysis of PFS indicated a correlation between PFS and cT, post-NACT SUVmax, pre-NACT MTV, post-NACT MTV, pre-NACT TLG, post-NACT TLG, the reduction rates for each PET-CT parameter, pre- and post-NACT PET-N, pT, pN, and histological grade (Table 3). Among preoperative factors, TLG reduction rate (HR = 2.58; 95% CI 1.16–5.73; *P* = 0.020) and post-NACT PET-N (HR = 1.79; 95% CI 1.04–3.08; *P* = 0.035) were significant independent prognostic factors in a multivariate analysis of PFS (multivariate model #1, Table 3). In addition, the TLG reduction rate (HR = 2.51; 95% CI 1.14–5.51; *P* = 0.022) and pN (HR = 1.96; 95% CI 1.24–3.10; *P* = 0.004) were both significant in the multivariate analysis of all factors for PFS (multivariate model #2, Table 3). Lastly, a Kaplan–Meier analysis of PFS for all patients classified into four groups based on TLG reduction and post-NACT PET-N, both of which were independent prognostic factors in the multivariate analysis (model #1), is shown in Fig. 3a. The same analysis of PFS for all patients classified based on TLG reduction and pN, both of which were independent prognostic factors in the multivariate analysis (model #2), is shown in Fig. 3b. The 5-year PFS rates were 60.7% among post-NACT PET-N (0–1)/TLG-responders, 36.7% among post-NACT PET-N (0–1)/TLG-non-responders, 33.3% among post-NACT PET-N (≥2)/TLG-responders, and only 9.1% among post-NACT PET-N (≥2)/TLG-non-responders (Fig. 3a). Furthermore, the 5-year PFS rates were 76.3% among pN(−)/TLG-responders, 50.0% among pN(−)/TLG-non-responders, 45.7% among pN(+)/TLG-responders, and 26.3% among pN(+)/TLG-non-responders (Fig. 3b).

**Table 3:** Univariate and multivariate survival analysis of progression-free survival.

		Univariate			Multivariate: model #1			Multivariate: model #2		
		HR	95% CI	*P* value	HR	95% CI	*P* value	HR	95% CI	*P* value
Age	<67/≥67	1.22	0.85–1.75	0.29						
Sex	Male/female	1.74	0.91–3.33	0.094						
Tumour location	Ut/Mt, Lt	1.08	0.66–1.76	0.77						
Chemotherapy regimen	Others/DCF	1.31	0.89–1.92	0.17						
cT	3–4/0–2	1.99	1.28–3.11	**0.0023**	1.09	0.61–1.96	0.76	0.95	0.52–1.71	0.85
cN	1–3/0	1.48	0.96–2.29	0.079						
SUVmax pre	≥12.79/<12.79	1.36	0.95–1.96	0.094						
SUVmax post	≥4.52/<4.52	2.33	1.62–3.36	**<0.0001**	1.65	0.83–3.28	0.15	1.58	0.77–3.26	0.21
SUVmax reduction rate	<60% />60%	1.75	1.21–2.53	**0.0027**	1.66	0.88–3.12	0.12	1.53	0.81–2.90	0.19
MTV pre	≥22.8/<22.8	1.64	1.14–2.36	**0.0074**	1.07	0.67–1.71	0.78	1.06	0.66–1.69	0.82
MTV post	≥3.20/<3.20	1.81	1.25–2.60	**0.0015**	0.51	0.12–2.20	0.37	0.51	0.12–2.23	0.37
MTV reduction rate	<80%/>80%	1.70	1.18–2.45	**0.0047**	0.51	0.22–1.17	0.11	0.49	0.22–1.14	0.097
TLG pre	≥42.2/<42.2	1.81	1.16–2.82	**0.0088**	1.91	0.89–4.08	0.096	1.74	0.81–3.71	0.15
TLG post	≥11.2/<11.2	1.95	1.35–2.81	**0.0003**	1.37	0.31–6.00	0.67	1.07	0.24–4.71	0.93
TLG reduction rate	<80%/>80%	2.04	1.41–2.97	**0.0002**	**2.58**	**1.16–5.73**	**0.020**	**2.51**	**1.14–5.51**	**0.022**
Number of PET-positive LNs before NACT	≥2 /0–1	1.66	1.15–2.39	**0.0069**	1.37	0.90–2.09	0.14	1.29	0.84–1.99	0.24
Number of PET-positive LNs after NACT	≥2/0–1	2.32	1.48–3.65	**0.0003**	**1.79**	**1.04–3.08**	**0.035**	1.68	0.97–2.92	0.063
Postoperative complication^a^	III–V/0–II	1.28	0.87–1.89	0.22						
pT	3–4/0–2	2.39	1.65–3.45	**<0.0001**				1.54	0.97–2.46	0.069
pN	1–3/0	2.44	1.57–3.80	**<0.0001**				**1.96**	**1.24–3.10**	**0.004**
Histological grade	0–1a/1b-3	1.71	1.18–2.47	**0.0044**				1.34	0.90–1.99	0.16

*DCF* docetaxel, cisplatin, 5-fluorouracil, *MTV* metabolic tumour volume, *TLG* total lesion glycolysis, *LN* lymph node, *NACT* neoadjuvant chemotherapy.

^a^Postoperative complication: classified by Clavien–Dindo classification.

Bold values indicate *P* values with stratistical significance

## Discussion

In the ROC analysis of recurrence and multivariate analysis of survival, the TLG reduction in the primary tumour during NACT was the best marker among the PET-CT parameters that we examined. Furthermore, a TLG reduction rate of 80% was the optimal cut-off to clearly discriminate between responders and non-responders to NACT based on the stepwise analysis and its correlation with pathological response. Multivariate survival analysis demonstrated that TLG reduction and post-NACT PET-N are independent prognostic parameters, suggesting the potential utility of a PET-based treatment strategy in advanced OSCC patients undergoing NACT plus surgery.

MTV and TLG have been reported to be better prognostic factors for survival than SUV in several types of cancer [39, 40]. Furthermore, in radiotherapy or preoperative CRT for advanced OC, TLG was recently reported to be superior to SUV and MTV in predicting the prognosis [41, 42]. Consistent with these reports, we have shown that the TLG reduction rate is only an independent prognostic parameter in patients with advanced OSCC who undergo surgery after NACT. In a multivariate analysis of MTV and TLG on PET-CT, Choi et al. previously reported that TLG was a more useful prognostic marker before surgical resection of sarcoma [43]. Other studies have also reported that TLG is the best marker in several types of cancer [44–47]. The reasons are speculated to be as follows. Though MTV represents only the amount of active metabolic tumour cells, TLG, calculated by multiplying the tumour volume by the SUVmean of the tumour, can provide more detailed information about the pathology than other PET-CT parameters because it reflects both biological features and total tumour volume throughout the body [44–46]. However, whether MTV or TLG is more useful in predicting prognosis and determining treatment efficacy is still controversial. As these factors are strongly related, it is difficult to compare which is the better marker, and there is no standardised method for comparing these parameters [15]. Therefore, the results of this study should be validated by another cohort in a future prospective study.

The advantage of the present study is a large number of cases compared to previous, similar PET-CT studies [23]. To the best of our knowledge, no previous report has shown the prognostic value of TLG reduction rate before and after NACT in such a large number of OSCC patients. Furthermore, all patients in this study had squamous cell carcinoma treated with NACT with a triplet regimen followed by surgery, whereas treatment methods were not standardised in many previous studies. Moreover, by directly comparing the correlations between the three PET-CT indices and clinico-pathological indices using the optimal cut-offs obtained by the stepwise analysis, a TLG reduction rate of 80% was the best index. Among preoperative parameters, post-NACT PET-N and the TLG reduction rate were identified as independent prognostic factors in a multivariate analysis of survival. This result suggests that PET-CT evaluation of both the primary tumour and LNs can preoperatively select patients with extremely poor prognosis who may benefit from additional chemotherapy with a different regimen or chemoradiation instead of immediate surgery. Goodman et al. also reported that changing chemotherapy regimens improved prognosis in oesophageal cancer patients with small reductions in SUVmax by PET-CT before and after induction chemotherapy [8]. In other words, early stratification of patients with poor response to treatment by PET-CT would be extremely important. Furthermore, these patients at high risk of recurrence may be good candidates for adjuvant therapy with immune checkpoint inhibitors, especially based on the results of the recent CheckMate577 trial [48]. Treatment strategies for the stratified patients with poor prognosis are an urgent issue for future study. Thus, it is notable that PET-CT during NACT, which is currently the standard treatment for advanced OSCC, may be better utilised to identify patients with poor prognosis at an early stage of multimodal treatment. Although TLG reduction is an indicator focusing only on the primary tumour, it was found to be a more accurate prognostic marker when combined with the post-NACT PET-N or pN stage. As the primary tumour and metastatic LNs behave differently during chemotherapy [7, 25, 35, 37], it would be of interest to evaluate changes in PET-CT parameters in metastatic LNs, combined or compared with the changes in the primary tumour.

This study has some limitations. First, this study is a retrospective investigation at a single institution, though a large cohort of patients was analysed. Second, we used a SUVmax of 2.5 as an absolute threshold for measuring MTV. There are two ways to define MTV, the absolute SUV threshold method and the fixed % SUVmax threshold method, but which method is better has not been decided. No statistical difference has been reported between the two methods [44]. The present results need to be verified in the future in a separate cohort. Third, this study included patients who received FAP and DCF therapy as NACT. Subgroup analysis by NACT may be necessary in the future.

In conclusion, the present study demonstrated that the TLG reduction during NACT, in addition to post-NACT PET-N, is useful for accurately predicting prognosis in advanced OSCC patients undergoing surgery following NACT.

## Supplementary information

Supplemental Information

## Data Availability

The data sets generated and/or analysed during the current study are available from the corresponding author on reasonable request.
