# Supplementary material for: Risk stratification of oesophageal squamous cell carcinoma using change in total lesion glycolysis and number of PET-positive lymph nodes
Source: Br J Cancer. 2023 Feb 25;128(10):1879–87. doi: 10.1038/s41416-023-02151-y (PMC10147681; doi:10.1038/s41416-023-02151-y)
Supplement: Supplementary file 1 — Supplemental Information [file 41416_2023_2151_MOESM1_ESM.docx]

**Supplemental figure 1. The measurement of PET-CT parameters**

Semi-quantitative and volumetric analysis method of the primary tumor using software SYNAPSE VINCENT^®^ to measure PET-CT parameters.

**Supplemental Table 1. Patient characteristics (n=226)**

| Age | median (range) | 67 (35-82) |
| --- | --- | --- |
| Sex | Male / Female | 197 / 29 |
| Tumor location | Upper / Middle / Lower | 35 / 101 / 90 |
| cT | 0 / 1 / 2 / 3 / 4 | 0 / 9 / 56 / 161 / 0 |
| cN | 0 / 1 / 2 / 3 | 62 / 125 / 37 / 2 |
| cM | 0 / 1 | 203 / 23 |
| cStage | I / II / III / IV | 0 / 94 / 109 / 23 |
| Regimen of preoperative chemotherapy | FAP* / DCF* / Others* | 65 / 157 / 4 |
| Surgical procedure | Open / VATS* / non-thoracotomy | 128 / 92 / 6 |
| Lymph nodes dissection | 2-field / 3-field | 97 / 129 |
| Number of LNs* dissected | median (range) | 58 (20-154) |
| Residual tumor | R0 / R1 / R2 | 220 / 6 / 0 |
| Postoperative complication* | 0 / I / II / III / IV / V | 87 / 20 / 56 / 53 / 10 / 0 |
| pT | 0 / 1 / 2 / 3 / 4 | 26 / 63 / 35 / 101 / 1 |
| pN | 0 / 1 / 2 / 3 | 75 / 73 / 51 / 27 |
| pM | 0 / 1 | 211 / 15 |
| pStage | 0 / I / II / III / IV | 19 / 35 / 58 / 99 / 15 |
| Histological response | Grade 0 / 1a / 1b / 2 / 3 | 3 / 77 / 62 / 59 / 25 |

FAP*: doxorubicin, cisplatin, 5-fluorouracil, DCF*: docetaxel, cisplatin, 5-fluorouracil, Others*: 5-fluorouracil and cisplatin (three patients), nedaplatin and paclitaxel (one patient) VATS*: video-assisted thoracoscopic surgery, LNs*: lymph nodes, Postoperative complication*: classified by Clavien-Dindo classification
